# Comparative Efficacy and Safety of Tirzepatide Versus Dulaglutide in Patients with Type 2 Diabetes Mellitus: A Systematic Review and Meta-Analysis

**DOI:** 10.3390/healthcare14070850

**Published:** 2026-03-27

**Authors:** Sadia Qazi, Mohammad Dawar Zahid, Eshal Atif, Anushah Faheem Ilyas, Mazhar Ali, Umair Ali, Muhammad Junaid, Eshal Fatima, Safia Bibi, Rai Muhammad Hassan Ashraf, Muhammad Atif Mazhar

**Affiliations:** 1 Department of Anatomy, College of Medicine, Alfaisal University, Riyadh 11533, Saudi Arabia; 2 Medical College, Aga Khan University, Karachi 74800, Pakistan; mohammaddawar.zahid@scholar.aku.edu (M.D.Z.); eshalfatima73457@gmail.com (E.F.); 3 College of Medicine, Alfaisal University, Riyadh 11533, Saudi Arabia; eatif@alfaisal.edu; 4 Karachi Medical and Dental College, Karachi Metropolitan University, Karachi 74700, Pakistan; anushah.faheem@gmail.com; 5 Khyber Medical College, Peshawar University, Peshawar 25120, Pakistan; mazharaliuss@gmail.com (M.A.); junaidkhanx55@gmail.com (M.J.); 6 Department of Pharmacy, University of Swabi, Swabi 23570, Pakistan; umairaliuoswabi@gmail.com; 7 Quetta Institute of Medical Sciences, Quetta 87300, Pakistan; safyakhan877@gmail.com; 8 Shifa College of Medicine, Shifa Tameer-e-Millat University, Islamabad 44000, Pakistan; rai.hassan.ashraf@gmail.com

**Keywords:** Type 2 diabetes mellitus, tirzepatide, dulaglutide, adverse events, treatment discontinuation, meta-analysis, GRADE

## Abstract

**Background:** Tirzepatide, a dual GIP/GLP-1 receptor agonist, demonstrates substantial glycemic and weight benefits versus GLP-1 receptor agonists in indirect comparisons, but direct comparative safety evidence versus dulaglutide remains limited. We evaluated comparative safety (primary outcome: overall adverse events) and efficacy. **Methods:** Following PRISMA 2020 (prospectively registered: PROSPERO CRD420251276594), we searched MEDLINE, Embase, Scopus, and CENTRAL (inception–31 December 2025) for randomized controlled trials (≥26 weeks) comparing once-weekly tirzepatide with dulaglutide in adults with type 2 diabetes. Three trials (N = 13,590 participants) were included. Dichotomous outcomes were pooled using random-effects models (risk ratios [RRs], 95% confidence intervals [CIs]). GRADE assessed certainty of evidence. **Results:** Overall adverse event incidence did not differ significantly (RR 1.04 [0.98–1.10]; I^2^ = 36%; moderate-certainty evidence). Discontinuation due to adverse events was consistently higher with tirzepatide (RR 1.32 [1.20–1.45]; I^2^ = 0%; high-certainty evidence), representing a 32% increased risk across all populations. Categorical HbA1c target achievement was analyzed in two trials; the third trial reported HbA1c as a continuous outcome only. At the primary threshold (HbA1c < 7.0%), tirzepatide was consistently superior with no heterogeneity (RR 1.48 [1.33–1.64]; I^2^ = 0%; *p* < 0.00001). Across all thresholds combined, heterogeneity was extreme (I^2^ = 92%), limiting confidence in any pooled summary estimate; the greatest instability occurred at the strictest threshold (HbA1c < 5.7%; I^2^ = 98%; *p* = 0.40). Tirzepatide showed greater HbA1c target attainment in treatment-naive patients receiving dulaglutide 0.75 mg, whereas the glycemic advantage was smaller in patients with established cardiovascular disease receiving dulaglutide 1.5 mg. Categorical weight-loss outcomes were analyzed in two trials; tirzepatide was associated with greater weight-loss threshold achievement (RR 8.80 [4.04–19.17]; very low-certainty evidence), although interpretation is limited by substantial heterogeneity and restricted generalizability. Serious adverse events were not significantly different (RR 0.82 [0.47–1.43]; I^2^ = 42%). **Conclusions:** Overall adverse events were similar between treatments, but tirzepatide consistently increased discontinuation risk, indicating a clinically important tolerability-persistence trade-off. Glycemic efficacy was highly population-dependent: benefits were consistent at the primary HbA1c target (<7.0%; I^2^ = 0%) in early-stage disease, whereas the advantage was smaller in long-standing disease with established cardiovascular disease. Tirzepatide may be favored when glycemic or weight efficacy is prioritized in earlier-stage disease, provided tolerability is proactively managed. Dulaglutide remains appropriate when persistence is threatened by tolerability concerns or cardiovascular risk reduction is the primary goal.

## 1. Introduction

Type 2 diabetes mellitus (T2DM) is a chronic, progressive metabolic disorder characterized by hyperglycaemia driven by insulin resistance and progressive β-cell dysfunction. Its global prevalence continues to rise, and the condition remains a major contributor to preventable morbidity and mortality through microvascular (retinopathy, nephropathy, neuropathy) and macrovascular (atherosclerotic cardiovascular disease, heart failure, stroke) complications. Accordingly, achieving and maintaining effective glycemic control is central to reducing complication risk and improving long-term outcomes, particularly in individuals with long disease duration and high cardiovascular risk [1,2].

Over the past decade, incretin-based therapies, most notably glucagon-like peptide-1 receptor agonists (GLP-1 RAs) have become core components of contemporary T2DM management, owing to their glucose-dependent glycemic efficacy, favorable weight effects, and broad cardiometabolic profile. GLP-1 RAs can improve HbA1c while supporting clinically meaningful weight reduction, and several agents have demonstrated cardiovascular benefit or safety in high-risk populations. This has positioned incretin therapies as an appropriate second-line or add-on choice when oral therapies fail to sustain glycaemic targets, especially among patients in whom weight, cardiovascular disease, or hypoglycaemia risk shape treatment selection [3].

Dulaglutide is a once-weekly GLP-1 RA widely used in routine practice and supported by clinical trials demonstrating improved glycemic control with modest weight loss. It has also demonstrated cardiovascular safety and benefit in high-risk populations in the REWIND cardiovascular outcomes trial [4], reinforcing its role in T2DM patients with established atherosclerotic cardiovascular disease or multiple cardiovascular risk factors. However, as with the GLP-1 RA class, dulaglutide is commonly associated with gastrointestinal adverse events, and inter-individual variability in glycaemic and weight response is frequently observed, which may influence adherence, persistence, and treatment satisfaction [4,5].

Tirzepatide is a once-weekly dual glucose-dependent insulinotropic polypeptide (GIP) and GLP-1 receptor agonist, representing a mechanistically broader incretin approach. The SURPASS clinical trial program established tirzepatide as a highly effective glucose-lowering agent with substantial weight reduction compared with several active comparators. A plausible explanation for its magnitude of effect is that dual incretin receptor agonism yields complementary metabolic actions: enhancing glucose-dependent insulin secretion, suppressing inappropriate glucagon secretion, slowing gastric emptying (particularly early in treatment), and reducing appetite and energy intake. However, these potent metabolic effects come with tolerability considerations, gastrointestinal adverse events similar to or potentially exceeding those with GLP-1 RAs remain clinically relevant and may contribute to treatment discontinuation in some patients, which has important implications for real-world effectiveness [6,7,8]. More recently, Nicholls et al. [9] provided the first cardiovascular outcomes evidence for tirzepatide in a high-risk population with established ASCVD, enabling direct head-to-head comparison with dulaglutide 1.5 mg over a median four-year follow-up.

Despite strong evidence supporting each agent independently, synthesizing direct comparative evidence between tirzepatide and dulaglutide remains challenging. Head-to-head randomized trials differ substantially in comparator dulaglutide dose (0.75 mg in Japan versus 1.5 mg internationally, representing different treatment intensities), enrolled populations (ranging from treatment-naive patients with early-stage disease to those with long-standing diabetes and established cardiovascular disease), background therapy, and follow-up duration (weeks to years) [10,11]. Critically, treatment tolerability and persistence often overlooked in favor of mean glycemic or weight changes, may significantly impact real-world benefit, as therapies that cannot be continued cannot deliver durable outcomes. Outcome reporting is also not uniform across studies, particularly for categorical “target attainment” endpoints versus continuous changes in HbA1c and body weight, complicating efforts to generate clinically interpretable comparative evidence.

Therefore, this systematic review and meta-analysis aims to evaluate the comparative safety and efficacy of tirzepatide versus dulaglutide in adults with T2DM. The primary objective is to assess overall adverse event incidence as a broad measure of treatment safety burden. Secondary objectives include evaluation of treatment discontinuation due to adverse events (reflecting tolerability and persistence), serious adverse events, and categorical efficacy outcomes including glycemic target achievement and weight loss thresholds. By synthesizing direct head-to-head randomized evidence across diverse populations and treatment contexts, this review seeks to inform clinically relevant treatment selection that balances efficacy expectations against tolerability trade-offs and patient-specific priorities. Access and affordability are additional considerations: as of 2025, tirzepatide is not reimbursed under national formularies in most countries outside the United States, which bears directly on whether comparative efficacy data translate into actionable prescribing decisions.

## 2. Methods

### 2.1. Protocol Registration and Reporting Standard

This systematic review and meta-analysis was conducted in accordance with the PRISMA 2020 reporting guideline. A completed PRISMA 2020 checklist documenting the location of all reported items within this manuscript is provided in Supplementary Table S1 [12]. The study protocol was prospectively registered in PROSPERO [13] (CRD420251276594) and is publicly accessible at: https://www.crd.york.ac.uk/prospero/display_record.php?ID=CRD420251276594, accessed 31 December 2025. No amendments were made to the protocol after registration.

### 2.2. Literature Search Strategy

A comprehensive literature search was performed in MEDLINE (PubMed), Embase, Scopus, and the Cochrane Central Register of Controlled Trials (CENTRAL) from database inception to 31 December 2025 to identify head-to-head randomized controlled trials (RCTs) comparing tirzepatide versus dulaglutide in adults with type 2 diabetes mellitus (T2DM). The search strategy combined controlled vocabulary (e.g., MeSH/Emtree) and free-text terms related to “tirzepatide”, “dulaglutide”, and “type 2 diabetes”, without language restrictions.

Search Strategies by Database:

PubMed/MEDLINE:

((“Diabetes Mellitus, Type 2”[Mesh] OR “type 2 diabetes”[tiab] OR T2DM[tiab] OR NIDDM[tiab] OR “non insulin dependent”[tiab]) AND (tirzepatide[tiab] OR LY3298176[tiab] OR Mounjaro[tiab] OR Zepbound[tiab] OR “tirzepatide”

((“Diabetes Mellitus, Type 2”[Mesh] OR “type 2 diabetes”[tiab] OR T2DM[tiab] OR NIDDM[tiab] OR “non insulin dependent”[tiab]) AND (tirzepatide[tiab] OR LY3298176[tiab] OR Mounjaro[tiab] OR Zepbound[tiab] OR tirzepatide[nm]))

Cochrane CENTRAL:

((tirzepatide OR “LY3298176” OR mounjaro OR zepbound) AND (dulaglutide OR “LY2189265” OR trulicity) AND (“type 2 diabetes” OR T2DM OR “diabetes mellitus type 2”))

Embase:

(‘type 2 diabetes mellitus’/exp OR ‘type 2 diabetes’:ti,ab,kw OR t2dm:ti,ab,kw OR niddm:ti,ab,kw OR ‘non insulin dependent’:ti,ab,kw) AND (‘tirzepatide’/exp OR tirzepatide:ti,ab,kw OR ly3298176:ti,ab,kw OR mounjaro:ti,ab,kw OR zepbound:ti,ab,kw) AND (‘dulaglutide’/exp OR dulaglutide:ti,ab,kw OR ly2189265:ti,ab,kw OR trulicity:ti,ab,kw)

Scopus:

TITLE-ABS-KEY ((“type 2 diabetes” OR “diabetes mellitus type 2” OR t2dm OR niddm OR “non insulin dependent”) AND (tirzepatide OR “LY3298176” OR mounjaro OR zepbound) AND (dulaglutide OR “LY2189265” OR trulicity))

The search strategies included both generic names (tirzepatide, dulaglutide) and brand names (Mounjaro, Zepbound, Trulicity), as well as manufacturer study codes (LY3298176, LY2189265) to maximize search sensitivity.

### 2.3. Eligibility Criteria

We included parallel-group RCTs published as full-text manuscripts that: (i) enrolled adults (≥18 years) with confirmed T2DM; and (ii) reported follow-up of ≥26 weeks.

We excluded studies enrolling non-T2DM populations (e.g., obesity-only cohorts), unless T2DM-specific data were separately extractable. Studies were also excluded if they: used incorrect study designs (e.g., observational studies), reported only outcomes not specified in our protocol, were available only as conference abstracts without full-text publications, or enrolled duplicate populations already represented in included trials. A complete list of excluded studies with specific reasons is provided in Supplementary Table S2.

Intervention: Once-weekly subcutaneous tirzepatide, administered as fixed maintenance doses (e.g., 5 mg, 10 mg, 15 mg) or dose-escalation regimens titrated up to a maximum of 15 mg, consistent with trial protocols.

Comparator: Once-weekly subcutaneous dulaglutide. Because dulaglutide dosing differs by region, analyses were prespecified to be stratified by comparator dose (0.75 mg vs. 1.5 mg). The primary comparator definition was dulaglutide 1.5 mg (global standard). Trials using 0.75 mg (regional standard in Japan) were included in dose-stratified subgroup and/or sensitivity analyses rather than pooled unstratified with 1.5 mg trials.

### 2.4. Outcomes

#### 2.4.1. Primary Outcome

The primary outcome was overall adverse event incidence, defined as the proportion of participants experiencing at least one adverse event of any severity during the trial period. This outcome was selected to provide a comprehensive assessment of the overall safety burden associated with each treatment, encompassing both common tolerability issues and potentially rare serious events, thereby capturing the full spectrum of treatment-related safety signals across diverse populations and durations.

#### 2.4.2. Secondary Safety Outcomes

Secondary safety outcomes included:

Treatment discontinuation due to adverse events: Defined as permanent cessation of study medication attributed to adverse events, reflecting treatment tolerability from the patient perspective.

Serious adverse events (SAEs): Defined per ICH-GCP guidelines as events resulting in death, life-threatening conditions, hospitalization (or prolongation of existing hospitalization), persistent or significant disability/incapacity, congenital anomaly/birth defect, or other medically important events requiring intervention to prevent serious outcomes.

#### 2.4.3. Efficacy Outcomes

Efficacy outcomes included categorical endpoints assessed at the end of follow-up or the nearest reported trial timepoint:

Glycemic control: Proportion of participants achieving prespecified HbA1c targets (e.g., HbA1c < 7.0%, <6.5%, <5.7%), reflecting attainment of guideline-recommended glycemic goals.

Weight loss: Proportion of participants achieving categorical weight-loss thresholds (e.g., ≥5%, ≥10%, or ≥15% body weight reduction from baseline), consistent with clinically meaningful weight loss definitions.

#### 2.4.4. Outcome Data Format

All outcomes were extracted and analyzed in dichotomous format (events/total participants). Continuous outcomes (e.g., mean change in HbA1c or body weight) were summarized narratively because quantitative pooling was not feasible for three reasons. First, Frias et al. [11] reported continuous outcomes using a Bayesian hierarchical model with 80% credible intervals, which cannot be combined with the frequentist 95% confidence intervals used in the other two trials. Second, outcomes were assessed at different timepoints across trials (26 weeks, 52 weeks, and a median of 36 months). Third, variance measures differed across trials (80% credible interval, standard error of least-squares means, and 95% confidence interval of mean difference). Continuous outcome data are summarized in results, with its variance reporting. Accordingly, only dichotomous outcomes consistently reported across trials were synthesized quantitatively.

### 2.5. Study Selection

Two reviewers independently screened titles and abstracts using Rayyan (Rayyan Systems Inc., Cambridge, MA, USA, retrieved potentially eligible reports for full-text review, and determined final eligibility [14]. Disagreements were resolved by consensus; if needed, a third reviewer adjudicated.

### 2.6. Data Extraction

Two reviewers independently extracted data using a standardized, pre-piloted form. Extracted variables included study characteristics (first author, year, region, trial registration), baseline participant characteristics (age, sex, BMI, HbA1c, diabetes duration, cardiovascular disease history), intervention/comparator regimens (dose, titration scheme, follow-up duration), and outcome data.

For meta-analysis, outcomes were extracted in dichotomous format. For each arm, the number of participants experiencing the outcome and the total number of evaluable participants were recorded in n/N format. Safety outcomes were extracted from the population used in each trial’s primary safety analysis: the full intention-to-treat population in Nicholls et al. [9], the modified intention-to-treat population in Inagaki et al. [10], and the safety set (all randomized participants who received at least one dose of study drug) in Frias et al. [11]. Efficacy outcomes were extracted from the modified intention-to-treat or per-protocol populations as reported by the primary trial publications.

For multi-arm trials; Inagaki et al. [10] and Frias et al. [11], data were extracted from the tirzepatide 15 mg arm only, as this represents the highest approved maintenance dose and is the clinically most relevant comparison. The corresponding control arm data were used once per trial to avoid double-counting of shared control participants.

### 2.7. Risk of Bias Assessment

Risk of bias (RoB) was assessed independently by two reviewers using the Cochrane Risk of Bias 2 (RoB 2) tool across five domains: randomization process; deviations from intended interventions; missing outcome data; outcome measurement; and selection of the reported result. Each domain was rated as low risk, some concerns, or high risk, with an overall risk-of-bias judgment derived using the RoB 2 algorithm. Disagreements were resolved by consensus [15].

### 2.8. Certainty of Evidence Assessment

The certainty of evidence for each outcome was assessed using the Grading of Recommendations, Assessment, Development and Evaluations (GRADE) approach. Evidence certainty was rated as high, moderate, low, or very low based on consideration of risk of bias, inconsistency (heterogeneity), indirectness, imprecision, and publication bias. Ratings were downgraded when substantial concerns were identified in any domain. GRADE assessments were conducted independently by two reviewers with disagreements resolved by discussion.

### 2.9. Statistical Analysis

All analyses were conducted using Review Manager (RevMan), (The Cochrane Collaboration, Copenhagen, Denmark) [16]. For dichotomous outcomes, treatment effects were summarized as risk ratios (RRs) with 95% confidence intervals (CIs). Meta-analyses used a random-effects Mantel–Haenszel model to account for anticipated clinical and methodological heterogeneity. Statistical heterogeneity was quantified using the I^2^ statistic and Cochran’s Q test, with I^2^ values of 0–40%, 30–60%, 50–90%, and 75–100% representing low, moderate, substantial, and considerable heterogeneity, respectively. For outcomes with I^2^ ≥ 75%, pooled estimates were reported with appropriate caution and contextualized by study-specific results.

A two-sided *p*-value < 0.05 was considered statistically significant for overall treatment effects. Statistical significance of heterogeneity was assessed using the chi-squared test with *p* < 0.10 indicating significant heterogeneity.

### 2.10. Subgroup and Sensitivity Analyses

Prespecified subgroup analyses were conducted according to dulaglutide dose (0.75 mg vs. 1.5 mg) as the primary exploration of heterogeneity, addressing the regional dosing difference between SURPASS J-mono (0.75 mg, Japan) [10] and the other two trials (1.5 mg, global) [9,11]. Pooled estimates across all three trials are presented in the main analysis because between-dose subgroup testing did not demonstrate a statistically significant interaction for safety outcomes (*p* for interaction > 0.10). All-trial pooled estimates are reported alongside dose-stratified forest plots for transparency. For efficacy outcomes where the dose difference between comparator arms is most likely to influence between-trial differences in effect size, trial-level estimates are presented separately in addition to the pooled figure, and the extreme heterogeneity observed (I^2^ = 92% for HbA1c target achievement) is attributed in part to the comparator dose disparity and population differences rather than to the true treatment effect variation alone.

Subgroup differences were evaluated using the test for interaction, with *p* < 0.10 considered suggestive of a subgroup effect.

### 2.11. Publication Bias Assessment

Assessment of publication bias was planned using funnel plot visual inspection and statistical tests (e.g., Egger’s test) for outcomes with ≥10 studies. Because fewer than 10 studies were available for quantitative synthesis, formal statistical assessment of publication bias was not performed. However, funnel plots were generated for all pooled outcomes with ≥3 studies to visually inspect for asymmetry, recognizing that interpretation is limited with small numbers of studies and that asymmetry may reflect heterogeneity rather than publication bias.

## 3. Results

### 3.1. Study Selection

**Figure 1:**
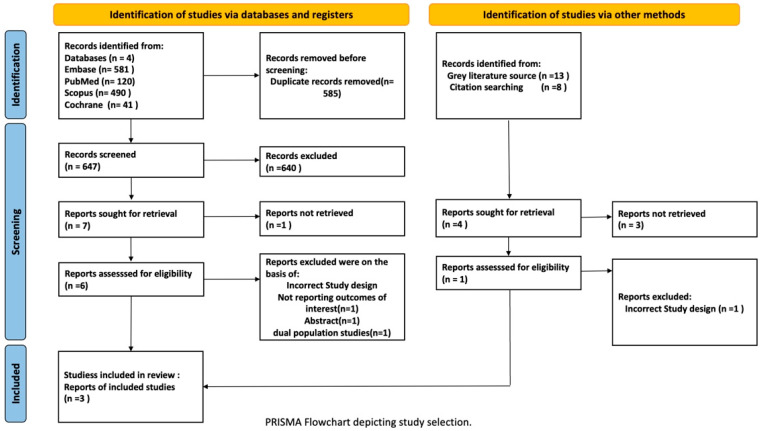
PRISMA Flowchart depicting the study selection process. The flowchart illustrates the systematic identification, screening, and inclusion of randomized controlled trials comparing tirzepatide 15 mg and dulaglutide.

The systematic search identified 1072 records from four databases (Embase, n = 581; PubMed, n = 120; Scopus, n = 490; Cochrane Library, n = 41) and other methods (gray literature sources, n = 13; citation searching, n = 8). After removal of 585 duplicate records, 647 unique records were screened by title and abstract, and 640 were excluded. Seven reports from databases and four reports from other methods were sought for retrieval. One database report and three reports from other methods were not retrieved. Six database reports and one report from other methods were assessed for eligibility. Four reports were excluded: three database reports due to incorrect study design (n = 1), not reporting outcomes of interest (n = 1), and incorrect dual population studies (n = 1), and one report from other methods due to incorrect study design (n = 1). A complete list of excluded studies with specific reasons for exclusion is provided in Supplementary Table S2. Ultimately, three randomized controlled trials, Nicholls et al. [9], Inagaki et al. [10], and Frias et al. [11], comprising 13,590 participants, were included in the systematic review and meta-analysis. The literature search was conducted from database inception through December 2025. See the PRISMA flow diagram (Figure 1).

### 3.2. Study Characteristics

The meta-analysis incorporated data from three randomized controlled trials (RCTs) comparing tirzepatide 15 mg with dulaglutide: Nicholls et al. [9], Inagaki et al. [10] and Frias et al. [11]. The Inagaki et al. [10] and Frias et al. [11] trials enrolled 318 participants total, while the Nicholls et al. [9] trial enrolled 13,165 participants.

Inagaki et al. [10] compared tirzepatide 15 mg to dulaglutide 0.75 mg, whereas Frias et al. [11] and Nicholls et al. [9] compared tirzepatide 15 mg to dulaglutide 1.5 mg. All three trials were double-blind RCTs with durations ranging from 26 to 52 weeks in efficacy trials to a median of 4.0 years in the cardiovascular outcomes trial (Table 1).

Risk of bias results across the five domains of the Cochrane RoB 2 tool are summarized in Supplementary Figure S1 and detailed in Supplementary Table S3. High risk in Inagaki et al. [10] reflects concerns regarding post hoc reporting of efficacy thresholds and missing data.

### 3.3. Baseline Characteristics

The three trials enrolled markedly different patient populations (Table 2). Mean age ranged from 56.3 years in Inagaki et al. [10] to 64.1 years in Nicholls et al. [9]. Female representation varied widely: 59% in Frias et al. [11], 28–29% in Nicholls et al. [9], and only 18% in Inagaki et al. [10]. Baseline HbA1c was similar across all arms (8.09–8.40%), but diabetes duration differed substantially, approximately 5 years in Inagaki et al. [10], 8.6 years in Frias et al. [11], and 14.7–14.8 years in Nicholls et al. [9]. All participants in Nicholls et al. [9] had established ASCVD; neither of the other two trials enrolled patients with cardiovascular disease history.

These differences define two clinically distinct profiles that directly shape the interpretation of all subsequent efficacy outcomes. Inagaki et al. [10] represents early-stage disease: treatment-naive or recently treated patients, lean BMI (mean 27.5 kg/m^2^), short diabetes duration (mean 5.0 years), no cardiovascular disease, and an exclusively Japanese cohort. Nicholls et al. [9] represents advanced disease: longstanding diabetes (mean 14.7 years), obesity (mean BMI 32.6 kg/m^2^), mandatory established ASCVD, 48.8% on insulin at baseline, and a multinational cohort with approximately 30% Hispanic/Latino participants across 30 countries. Pooled efficacy estimates should be interpreted with explicit reference to which profile a given patient most closely resembles.

### 3.4. Primary Outcome: Overall Adverse Events

**Figure 2:**
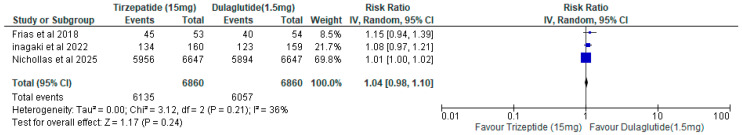
Forest plot of overall adverse events of three trials; Nicholls et al. [9], Inagaki et al. [10] and Frias et al. [11]. Risk ratios (RRs) comparing tirzepatide 15 mg with dulaglutide in adults with type 2 diabetes mellitus. Data are presented with 95% confidence intervals (CIs). Heterogeneity was assessed using the I^2^ statistic (I^2^ = 36%).

Across the included trials, 6135 of 6860 participants experienced any adverse event in the tirzepatide arm (89.4%) compared with 6057 of 6860 in the dulaglutide arm (88.3%). The denominator of 6860 reflects the combined safety populations: SURPASS-CVOT (tirzepatide n = 6647, dulaglutide n = 6647), SURPASS J-mono (tirzepatide 15 mg arm n = 159, dulaglutide n = 159), and Frias et al. [11] Phase 2b (tirzepatide 15 mg arm n = 53, dulaglutide n = 54). All safety outcomes were analyzed in the safety population, defined as all randomized participants who received at least one dose of study drug; a per-study denominator accounting table is provided in Supplementary Table S4. The pooled risk ratio (RR) was 1.04 (95% CI: 0.98 to 1.10; *p* = 0.24), with low-to-moderate heterogeneity (I^2^ = 36%; chi-squared = 3.12, df = 2; *p* = 0.21), indicating no significant overall difference in adverse event incidence between treatments (Figure 2).

### 3.5. Secondary Safety Outcomes

#### 3.5.1. Treatment Discontinuation Due to Adverse Events

**Figure 3:**
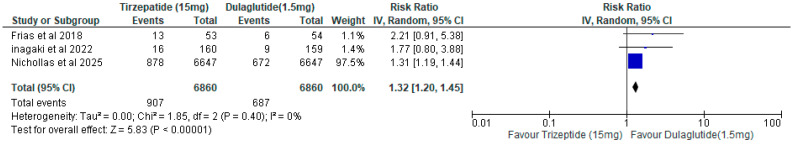
Forest plot of treatment discontinuation due to adverse events of three trials; Nicholls et al. [9], Inagaki et al. [10] and Frias et al. [11]. Analysis of permanent treatment cessation attributed to adverse effects across included trials. The pooled estimate demonstrates a consistently higher risk for tirzepatide (RR 1.32) with zero statistical heterogeneity (I^2^ = 0%).

Across all three trials, 907 of 6860 participants (13.2%) discontinued tirzepatide 15 mg due to adverse events versus 687 of 6860 participants (10.0%) receiving dulaglutide. The pooled risk ratio was 1.32 (95% CI: 1.20 to 1.45; *p* < 0.00001) with zero heterogeneity (I^2^ = 0%; chi-squared = 1.85, df = 2, *p* = 0.40). This represents the most internally consistent finding in this meta-analysis, with I^2^ = 0% confirming direction and magnitude of effect across all three trials. All safety populations were analyzed as treated (safety set), defined as all randomized participants who received at least one dose of the study drug, which differs from the modified intent-to-treat population used for efficacy analyses; this distinction is documented in Supplementary Table S4. The absolute risk difference was 3.1 percentage points in Nicholls et al. [9] (13.2% vs. 10.1%), corresponding to a number needed to harm of approximately 32 over a median follow-up of four years. In clinical terms, for every 32 patients treated with tirzepatide rather than dulaglutide, one additional patient is expected to discontinue treatment due to adverse events. Discontinuation was driven predominantly by gastrointestinal adverse events, which occurred in 42.5% of tirzepatide-treated and 35.9% of dulaglutide-treated participants [9] (Figure 3).

#### 3.5.2. Serious Adverse Events

**Figure 4:**
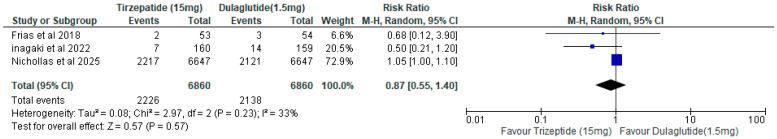
Forest plot of serious adverse events of three trials; Nicholls et al. [9], Inagaki et al. [10] and Frias et al. [11]. Comparison of incidences of life-threatening or medically significant events.

Serious adverse events occurred in 2226 of 6860 participants (32.4%) in the tirzepatide arm compared with 2138 of 6860 participants (31.2%) in the dulaglutide arm. The pooled risk ratio was 0.82 (95% CI: 0.47 to 1.43; *p* = 0.49), with low-to-moderate heterogeneity (I^2^ = 42%; chi-squared = 3.14, df = 2, *p* = 0.21). The wide confidence interval reflects the relatively small event counts in the two smaller trials [10,11] (combined n = 426) alongside the dominant contribution of (n = 13,294) [9], where serious adverse events were frequent and the estimate carried most of the statistical weight. Overall, these findings suggest no significant difference in serious adverse event risk between tirzepatide 15 mg and dulaglutide 1.5 mg, and the result should be interpreted primarily as evidence of no clear increase in risk rather than as a precise estimate of effect size (Figure 4).

### 3.6. Efficacy Outcomes

#### 3.6.1. Glycemic Control (HbA1c)

##### Heterogeneity Assessment

**Figure 5:**
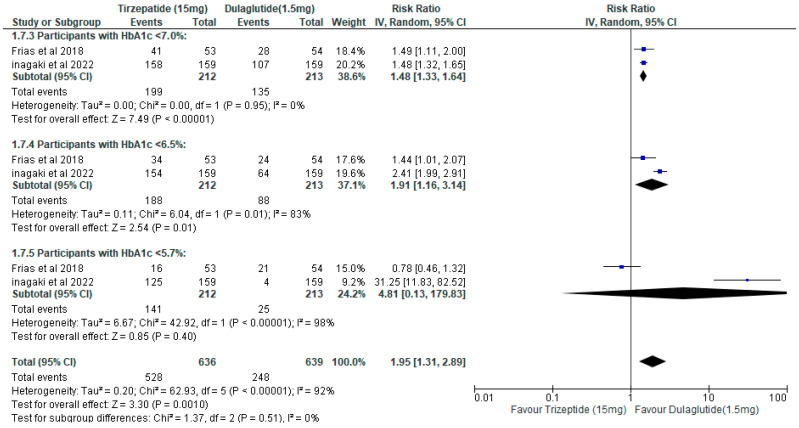
Forest plot of HbA1c target achievement comparing tirzepatide 15 mg versus dulaglutide across three prespecified thresholds (<7.0%, <6.5%, <5.7%), based on two trials [10,11]. Pooled using a random-effects inverse-variance model. Marked heterogeneity (overall I^2^ = 92%) reflects divergent trial-level estimates at stricter thresholds; the <7.0% primary threshold was homogeneous (I^2^ = 0%).

Pooled analysis of two randomized controlled trials demonstrated that tirzepatide 15 mg was more effective than dulaglutide 1.5 mg in achieving categorical HbA1c targets. The likelihood of achieving HbA1c < 7.0% was significantly higher with tirzepatide (RR 1.48, 95% CI 1.33–1.64; *p* < 0.00001; I^2^ = 0%). Similarly, tirzepatide significantly increased attainment of HbA1c < 6.5% (RR 1.91, 95% CI 1.16–3.14; *p* = 0.01), although heterogeneity was substantial (I^2^ = 83%). In contrast, no significant difference was observed for HbA1c < 5.7% (RR 4.81, 95% CI 0.13–179.83; *p* = 0.40; I^2^ = 98%). When all HbA1c threshold outcomes were combined, tirzepatide remained significantly superior to dulaglutide overall (RR 1.95, 95% CI 1.31–2.89; *p* = 0.001), but heterogeneity was high (I^2^ = 92%) (Figure 5).

#### 3.6.2. Weight Loss (Threshold Achievement)

**Figure 6:**
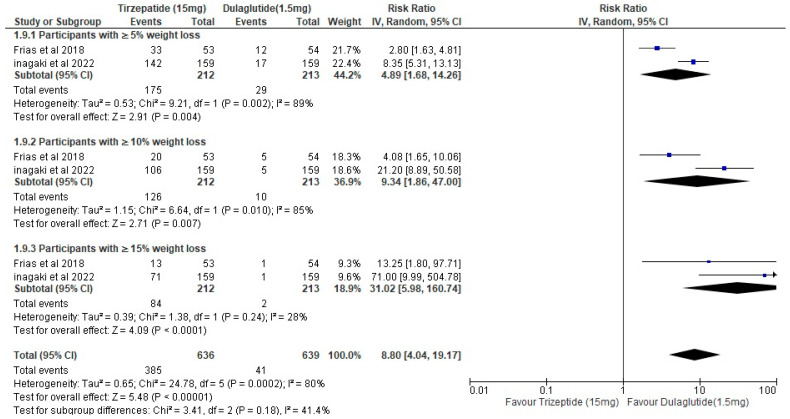
Forest plot of weight loss outcomes, based on two trials [10,11]. Likelihood of achieving body weight reduction thresholds of 5%, 10%, or 15% or more.

Two trials comprising 1275 participants overall (SURPASS J-mono, n = 318 [10]; Frias et al. Phase 2b [11], n = 107; with 850 additional participants from the pooled threshold analysis) were included in the categorical weight loss analysis. Tirzepatide 15 mg was associated with significantly higher rates of achieving all weight loss thresholds compared with dulaglutide, with the greatest relative effect observed at the 15% or greater threshold (RR 31.02, *p* < 0.0001). This analysis is substantially driven by SURPASS J-mono [10], which contributed the majority of participants. Weight loss data were not available from SURPASS-CVOT [9] in threshold-achievement format; continuous weight change data from that trial (tirzepatide −11.6% vs. dulaglutide −4.8%) are presented in Table 3. Given the absence of SURPASS-CVOT [9] weight threshold data and the differences in baseline BMI across trials (Inagaki [10]: 27.5 kg/m^2^ vs. Frias et al. [11]: 33.1–34.4 kg/m^2^), the pooled weight loss estimate should not be generalized to populations with higher baseline BMI or established cardiovascular disease. Heterogeneity was substantial (I^2^ = 80%), consistent with these population differences (Figure 6).

#### 3.6.3. Continuous Efficacy Outcomes and Heterogeneity Evidence

Quantitative pooling of continuous changes in HbA1c and body weight was precluded by non-uniform reporting and inconsistent variance measures. As shown in Table 3, tirzepatide 15 mg consistently demonstrated numerically greater reductions in both parameters compared with dulaglutide; detailed variance reporting and the reasons these outcomes could not be pooled are provided in Supplementary Table S5.

### 3.7. Publication Bias Assessment

Visual inspection of funnel plots was performed for outcomes with three or more data points (Supplementary Figures S2–S6).

Overall Adverse Events: The funnel plot showed symmetric distribution around the pooled estimate of RR 1.04 (Supplementary Figure S2).

Treatment Discontinuation: The plot clustered symmetrically around the pooled RR of 1.32 (Supplementary Figure S3).

Serious Adverse Events: The funnel plot showed symmetric distribution with no obvious asymmetry (Supplementary Figure S4).

Glycemic Control Outcomes: The funnel plot showed asymmetry (Supplementary Figure S5). Given the extreme heterogeneity (I^2^ = 92%) and the fundamental differences in populations, comparator doses, and outcome measurement approaches across trials, funnel plot asymmetry here is most parsimoniously explained by clinical and methodological heterogeneity rather than publication bias per se.

Weight Loss Outcomes: Funnel plot assessment for weight loss thresholds is presented in Supplementary Figure S6.

### 3.8. Summary of Findings

Table 4 summarizes all outcomes with GRADE certainty ratings.

The most robust finding is the consistently higher rate of treatment discontinuation with tirzepatide (RR 1.32, 95% CI 1.20–1.45; I^2^ = 0%; high certainty), corresponding to a number needed to harm of approximately 32 over four years, driven primarily by gastrointestinal adverse events. The consistent direction across all three trials, zero heterogeneity, large sample size, and low risk of bias in Nicholls et al. support high certainty [9]. Overall adverse event incidence did not differ significantly (RR 1.04, 95% CI 0.98–1.10; moderate certainty), downgraded one level for low-to-moderate heterogeneity and marginal imprecision at the lower confidence bound.

For serious adverse events, the pooled estimate was non-significant (RR 0.82, 95% CI 0.47–1.43; low certainty), downgraded two levels for imprecision—the confidence interval spans clinically meaningful harm and benefit—and moderate heterogeneity (I^2^ = 42%).

Efficacy outcomes were downgraded to very low certainty and should not be interpreted as generalizable treatment effects. The HbA1c pooled estimate (RR 1.95, 95% CI 1.31–2.89) was downgraded three levels for extreme heterogeneity (I^2^ = 92%), incompatible comparator doses (0.75 mg vs. 1.5 mg dulaglutide), and high risk of bias in Inagaki et al. [10] regarding efficacy threshold reporting. The weight loss estimate (RR 8.80, 95% CI 4.04–19.17) was downgraded three levels for extreme heterogeneity (I^2^ = 80%), single-trial dominance, and the complete absence of extractable categorical weight data from Nicholls et al. [9]. For both efficacy outcomes, multiple thresholds from each trial are presented separately in forest plots rather than pooled as independent observations, to avoid within-study correlation artifacts; the estimates in Table 4 represent overall meta-analytic summaries for reference only.

## 4. Discussion

This systematic review and meta-analysis synthesized evidence from three randomized controlled trials (N = 13,590) directly comparing tirzepatide 15 mg with dulaglutide in adults with type 2 diabetes mellitus (T2DM). The primary finding was that overall adverse event incidence did not differ significantly between treatments (RR 1.04, 95% CI: 0.98–1.10; moderate-certainty evidence). Treatment discontinuation due to adverse events was consistently higher with tirzepatide across all three populations (RR 1.32, 95% CI: 1.20–1.45; I^2^ = 0%; high-certainty evidence), representing the most internally consistent finding in this analysis. Efficacy analyses revealed extreme heterogeneity (I^2^ = 80–92%), reflecting fundamental differences in population characteristics, disease stage, comparator dose, and outcome measurement rather than sampling variation. Tirzepatide demonstrated numerically greater glycemic reductions across all three trials, but the magnitude and clinical interpretation differ substantially by context. These findings define a clinically important tolerability-persistence trade-off whose relevance varies by patient profile [17,18].

### 4.1. Primary Outcome: Overall Adverse Event Incidence

Overall adverse event incidence showed no statistically significant difference between tirzepatide and dulaglutide (RR 1.04, 95% CI: 0.98–1.10; *p* = 0.24), with low-to-moderate heterogeneity (I^2^ = 36%). This finding indicates that the broad safety burden is comparable between these agents at the population level, which is consistent with prior meta-analyses indicating that tirzepatide has an acceptable safety profile with event patterns broadly similar to GLP-1 receptor agonist-based therapies [19,20,21,22].

However, overall adverse event incidence is an inherently non-specific composite outcome that may obscure clinically meaningful differences in specific subtypes. The low-to-moderate heterogeneity (I^2^ = 36%) reflects some variability in population tolerance across trials without undermining the pooled estimate. Granular safety outcomes, including gastrointestinal adverse events by subtype and hypoglycemia rates, would provide greater clinical utility for treatment selection but were not uniformly reported across trials in formats suitable for meta-analysis.

### 4.2. Treatment Discontinuation: The Clinically Consequential Safety Signal

Despite similar overall adverse event rates, treatment discontinuation due to adverse events was significantly and consistently higher with tirzepatide (RR 1.32, 95% CI: 1.20–1.45; *p* < 0.00001), representing a 3.2 percentage point absolute increase in discontinuation risk. Critically, this finding demonstrated zero heterogeneity (I^2^ = 0%), indicating a consistent tolerability signal across diverse populations regardless of baseline body mass index, diabetes duration, ethnicity, or dulaglutide comparator dose. This uniformity suggests a treatment-inherent tolerability challenge rather than a population-specific phenomenon [17,20].

This finding is particularly consequential because discontinuation represents the point at which pharmacologic efficacy becomes irrelevant, a therapy that cannot be continued cannot deliver durable glycemic or weight benefit. The most plausible mechanism underlying this signal is dose-dependent gastrointestinal intolerance during medication escalation, including nausea, vomiting, early satiety, and reduced food intake, which while not classified as “serious” adverse events, significantly impact patients’ willingness to persist with therapy [17,18]. This interpretation is consistent with broader evidence syntheses showing that gastrointestinal adverse events are the primary driver of discontinuation with potent incretin-based therapies [19,20,21,22].

From a real-world effectiveness perspective, the higher discontinuation rate with tirzepatide partially offsets its superior on-treatment efficacy. Clinicians must therefore balance expected glycemic and weight benefits against the substantial risk that patients may not tolerate the medication long-term, particularly in populations with prior intolerance to GLP-1 receptor agonists or complex comorbidity profiles [21,22].

### 4.3. Glycemic Efficacy: Population-Specific Benefits and Analytical Limitations

#### 4.3.1. The Heterogeneity Problem

The pooled risk ratio for HbA1c target achievement was 1.95 (95% CI: 1.31–2.89; *p* < 0.00001) across two trials [10,11], with substantial heterogeneity (I^2^ = 92%). Nicholls et al. [9] reported HbA1c only as a continuous outcome and contributed no categorical data to this analysis. The heterogeneity reflects four compounding factors: (1) comparator dose heterogeneity (0.75 mg vs. 1.5 mg dulaglutide); (2) population heterogeneity (treatment-naive, lean Japanese patients vs. obese patients with established atherosclerotic cardiovascular disease); (3) outcome measurement heterogeneity (binary target achievement in two trials vs. continuous mean difference in the third); and (4) disease stage heterogeneity (median diabetes duration 5 years vs. 15 years). These differences represent distinct clinical questions being asked in distinct populations and are not amenable to resolution by statistical adjustment. Trial-level estimates, presented separately below and in Table 3, are the appropriate basis for clinical inference.

#### 4.3.2. Trial-Specific Findings

Inagaki et al. [10]: In treatment-naive Japanese patients with relatively short diabetes duration (mean 5 years) and lower BMI (27.5–28.0 kg/m^2^), tirzepatide 15 mg demonstrated marked superiority over dulaglutide 0.75 mg for achieving aggressive glycemic targets, with 158/159 versus 107/159 participants achieving HbA1c < 7.0%, and risk ratios ranging from 1.48 at HbA1c ≤ 7.0% to substantially larger effects at the strictest threshold (HbA1c ≤ 5.7%). However, three factors limit the generalizability of these findings: the comparator dose (0.75 mg) is the Japan-approved regional dose, representing half the internationally recommended 1.5 mg dose; the population was treatment-naive with likely preserved beta-cell function; and follow-up (52 weeks) does not reflect long-term persistence or efficacy.

Frias et al. [11]: In this dose-finding trial, 41/53 tirzepatide-treated versus 28/54 dulaglutide-treated participants achieved HbA1c < 7.0% at 26 weeks. These results are hypothesis-generating rather than confirmatory, as the trial used a Bayesian hierarchical model with 80% credible intervals, was conducted in a mixed international population (Poland, Puerto Rico, Slovakia, USA) with a suboptimal 6-week titration schedule and was underpowered for safety conclusions. The authors themselves characterized the study as hypothesis-generating.

Nicholls et al. [9]: In obese patients with established atherosclerotic cardiovascular disease and long-standing diabetes (mean duration 14.7 years), tirzepatide 15 mg produced a 0.78 percentage point greater reduction in HbA1c than dulaglutide 1.5 mg at 36 months (difference −0.78 pp, 95% CI: −0.84 to −0.72). This is a statistically significant and directionally consistent glycemic advantage. However, the magnitude is more modest than that seen in Inagaki et al. [10], and the higher discontinuation rate with tirzepatide (13.2% vs. 10.1%) means that the population-level glycemic benefit in intention-to-treat analyses is attenuated by those who did not complete treatment.

This discordance in magnitude across trials likely reflects multiple mechanisms: progressive beta-cell exhaustion in long-standing disease may reduce incremental benefit from dual incretin agonism; the high-risk Nicholls et al. [9] population was already receiving intensive background therapy, creating a ceiling effect; and longer follow-up (median 4 years vs. 52 weeks) captures disease progression and medication fatigue that attenuate initial benefits.

#### 4.3.3. Contextualization with Broader Evidence

The directional finding that tirzepatide demonstrates greater glycemic efficacy than GLP-1 receptor agonists is concordant with recent systematic reviews and network meta-analyses [19,20,21,22]. However, those syntheses typically rely on indirect comparisons, shorter follow-up durations, and populations enriched for treatment responsiveness. The present analysis adds the observation that in populations with treatment-refractory disease, long diabetes duration, and established cardiovascular disease, the glycemic advantage of tirzepatide over standard-dose dulaglutide is smaller and must be weighed against differential discontinuation rates.

From a mechanistic standpoint, dual GIP/GLP-1 receptor agonism plausibly provides additive metabolic effects including enhanced glucose-dependent insulin secretion, suppressed glucagon, and appetite reduction that contribute to weight loss and may secondarily improve glycemia [20,22]. However, mechanistic plausibility does not guarantee proportional clinical benefit across all populations, and the Nicholls et al. [9] data indicate that context-dependence is substantial.

### 4.4. Weight Loss: Substantial Benefits with Methodological Caveats

The categorical weight loss analysis showed a large, pooled effect favoring tirzepatide (RR 8.80, 95% CI: 4.04–19.17; I^2^ = 80%), with the largest relative effect at the ≥15% threshold (RR 31.02). However, this finding requires significant qualification. Weight loss data in threshold-achievement format were available from Inagaki et al. [10] and Frias et al. [11] only; Nicholls et al. [9], which enrolled 13,165 participants, reported weight change as a continuous percentage and was not included in this analysis. The pooled estimate is therefore driven by a single confirmatory trial (Inagaki et al. [10], N = 318) conducted in a lean Japanese population (mean baseline BMI 27.5 kg/m^2^) against the Japan-approved regional dulaglutide dose (0.75 mg). The substantial heterogeneity (I^2^ = 80%) is consistent with these differences and reinforces that this estimate should not be generalized to obese populations receiving standard-dose comparators.

The directional conclusion that tirzepatide produces greater weight reduction than dulaglutide is consistent with broader evidence syntheses [19,20,21,22] and with the continuous weight data from Nicholls et al. [9] (−11.6% vs. −4.8%). This difference is clinically relevant for patients with obesity-related complications or those for whom weight reduction is a primary therapeutic goal alongside glycemic control. Confirmatory threshold-achievement data from large trials using standard comparator doses are still absent and represent a gap in the current evidence base.

### 4.5. Serious Adverse Events: Conflicting Signals Require Cautious Interpretation

The pooled analysis of serious adverse events showed no statistically significant difference (RR 0.82, 95% CI: 0.47–1.43; *p* = 0.49; low-certainty evidence). The wide confidence interval reflects the small event counts in the two smaller trials [10,11], (combined n = 425) against the dominant contribution of Nicholls et al. [9] (n = 13,294). In Nicholls et al. [9] specifically, serious adverse event incidence was nearly identical between groups (31.8% tirzepatide vs. 31.9% dulaglutide), confirming no clinically meaningful difference in the largest and most rigorous trial. The pooled estimate should therefore be interpreted as evidence of no increased serious adverse event risk with tirzepatide rather than as a precise magnitude estimate. Directional inconsistency between smaller and larger trials in this outcome is most parsimoniously explained by small-sample variability rather than a true population-specific effect.

### 4.6. The Paradox of Metabolic Superiority Without Cardiovascular Benefit

A critical contextual finding from Nicholls et al. [9] that warrants explicit discussion is the absence of cardiovascular superiority for tirzepatide despite demonstrated metabolic benefits. The trial met its primary endpoint of non-inferiority for the composite of cardiovascular death, myocardial infarction, or stroke (HR 0.92, 95.3% CI: 0.83–1.01; *p* = 0.003 for non-inferiority; *p* = 0.09 for superiority), but did not demonstrate superiority, despite tirzepatide producing approximately 0.78 percentage points greater HbA1c reduction and 6.8 percentage points greater relative weight loss over a median of four years.

This dissociation between metabolic efficacy and cardiovascular outcomes carries three clinical implications. First, incremental glucose-lowering and weight reduction beyond what is achieved with standard-dose GLP-1 receptor agonists do not translate proportionally into cardiovascular risk reduction in this high-risk population. Second, cardiovascular benefit with incretin-based therapies likely derives substantially from mechanisms beyond glycemic control, including direct vascular and anti-inflammatory effects, blood pressure reduction, and natriuresis, for which there is no evidence of differential advantage for dual versus single incretin agonism. Third, the higher discontinuation rate observed with tirzepatide in Nicholls et al. [9] may itself attenuate population-level cardiovascular benefit in intention-to-treat analyses, even where on-treatment metabolic effects are superior.

For patients in whom cardiovascular risk reduction is the primary treatment goal, dulaglutide’s established cardiovascular outcomes trial evidence from REWIND [4] makes it a well-supported option. Tirzepatide’s role in this context is non-inferior but not yet superior, and the clinical value of its additional glycemic and weight benefits must be balanced against its tolerability profile.

### 4.7. Heterogeneity, Generalizability, and the Limits of Pooled Estimates

The marked between-study heterogeneity in this meta-analysis reflects meaningful clinical and methodological differences rather than statistical noise. Age and disease duration ranged from middle-aged treatment-naive patients (mean age 56 years, diabetes duration 5 years) to older adults with long-standing treatment-refractory disease (mean age 64 years, duration 15 years). BMI ranged from overweight (27.5 kg/m^2^) to obese (32.6 kg/m^2^) populations with distinct metabolic phenotypes [21]. Cardiovascular risk ranged from primary prevention cohorts with no documented cardiovascular disease to 100% secondary prevention populations with mandatory established atherosclerotic disease. Comparator dose varied between the Japan-approved 0.75 mg and the internationally standard 1.5 mg dulaglutide. Follow-up ranged from 26 weeks to a median of four years.

These factors define distinct clinical populations [22], not variations around a single average. Consequently, treatment effects should be interpreted within the context of the enrolled population rather than applied universally. This heterogeneity is also consistent with broader meta-analytic evidence showing that relative treatment effects vary by baseline risk, concomitant therapy, and endpoint definition even when directionality is stable [18,19,20,21,22].

### 4.8. Clinical Implications and Treatment Selection

These findings support tirzepatide as a reasonable therapeutic choice when the clinical priority is achieving aggressive glycemic targets or substantial weight reduction in patients with earlier-stage disease, lower BMI, or treatment-naive status [20,22]. Clinicians must balance these benefits against the consistent signal of higher discontinuation risk and should manage gastrointestinal adverse events proactively during dose escalation. Practical strategies include structured titration protocols, early follow-up contacts, patient education regarding the expected symptom trajectory, and predefined criteria for temporary dose reduction or switching [17].

Dulaglutide may be preferred in several clinical contexts [21]: patients with prior intolerance to potent incretin therapies; older adults with complex comorbidity in whom polypharmacy and frailty threaten persistence; patients in whom cardiovascular risk reduction is the predominant goal, given REWIND’s established outcomes data [4]; and patients with long-standing, treatment-refractory diabetes in whom the incremental glycemic benefit of dual incretin agonism may be limited.

Cost and access are practical considerations that bear directly on whether these comparative data translate into clinical decisions. As of 2025, tirzepatide is not reimbursed under national formularies in most countries outside the United States, and the per-patient annual cost significantly exceeds that of dulaglutide in most health systems. The superior efficacy demonstrated in this analysis must therefore be contextualized against real-world access constraints: for most patients globally, dulaglutide remains the more accessible and cost-effective option irrespective of comparative efficacy estimates. Future analyses should incorporate cost-effectiveness modeling to guide formulary decisions.

At a fundamental level, these findings reinforce that the optimal therapy is not necessarily the one with the largest mean HbA1c reduction in clinical trials, but the one that individual patients can tolerate, persist with, and derive net benefit from over time. Shared decision-making should address expected efficacy, early adverse effects, discontinuation risk, patient preferences regarding side effects and injection burden, and the trade-offs inherent in selecting more potent versus more tolerable therapies [22].

### 4.9. Implications for Healthcare Delivery and Medication Management

The consistent discontinuation gap favoring dulaglutide in tolerability-persistence terms shifts the treatment selection framework from a “most potent agent” model to a “best sustainable agent” model. In chronic disease management, continuity is a determinant of outcome: a therapy that is not sustained cannot deliver durable glycemic or weight benefit regardless of on-treatment efficacy.

Medication management should therefore include a predefined persistence plan at treatment initiation. For tirzepatide, this plan should specify early monitoring during dose escalation, rapid access for adverse-effect management, and clear criteria for dose adjustment or switching. This approach keeps patient safety central while improving treatment continuity.

### 4.10. Care-Pathway Implications of the Discontinuation Signal

The discontinuation finding (RR 1.32; I^2^ = 0%) supports pathway-level adjustments. A pragmatic care pathway may include: (1) early follow-up shortly after initiation and after each dose escalation step; (2) structured titration support with symptom checklists and hydration and nutrition guidance; (3) standardized adverse-event counseling before the first dose, including expected symptom trajectory; (4) predefined mitigation algorithms including temporary dose hold, slower up-titration, or switch criteria; and (5) documentation of tolerability milestones as part of routine quality monitoring. These actions are intended to reduce avoidable discontinuation and should be interpreted as implementation-oriented guidance informed by, but not proven causal from, this meta-analysis.

### 4.11. Implementation Relevance: Persistence, Real-World Continuity, and Patient-Centered Decision-Making

Implementation relevance is strongest when trial findings are translated into service metrics that matter in routine care. For incretin-based therapies, persistence and continuity should be treated as co-primary practical endpoints alongside HbA1c and weight outcomes. Programs may track early discontinuation rates, dose-escalation completion, unscheduled intolerance visits, and therapy continuity at prespecified intervals to assess real-world quality performance.

Treatment choice should reflect individual priorities including glycemic intensity targets, weight goals, side-effect tolerance, follow-up capacity, and prior intolerance history, rather than a single pooled efficacy estimate. In this framework, safety and persistence are not secondary considerations; they are central determinants of net clinical benefit.

### 4.12. Limitations

This review has several limitations that affect the interpretation and generalizability of findings. Only three randomized controlled trials met inclusion criteria, and pooled safety analyses were dominated by a single large trial Nicholls et al. [9] contributing >97% of statistical weight for discontinuation, which may mask smaller-study signals. Substantial between-study heterogeneity in population characteristics, comparator dosing, and outcome definitions limited the validity of pooled estimates for efficacy outcomes, where extreme heterogeneity (I^2^ = 80–92%) rendered the overall pooled effect unreliable for clinical decision-making.

Critical safety outcomes were not available for meta-analysis, including hypoglycemia by severity grade, gastrointestinal adverse events by subtype, and reasons for discontinuation beyond adverse events. These granular outcomes are essential for individualized therapy selection but were either not uniformly reported or reported in formats precluding quantitative synthesis. Continuous outcomes could not be pooled due to incompatible statistical frameworks (Bayesian credible intervals vs. frequentist confidence intervals), non-uniform timepoints, and incomplete variance data.

Weight loss data were available from only two trials, with the pooled estimate substantially driven by Inagaki et al. [10] (N = 318) in a lean Japanese population using the Japan-approved regional dulaglutide dose (0.75 mg); the pooled analysis also combined multiple thresholds from the same population, which inflates apparent heterogeneity and effect sizes. Cardiovascular outcomes were not evaluated, as only one trial Nicholls et al. [9] was designed as a cardiovascular outcomes trial and indirect comparisons would be methodologically inappropriate. Long-term outcomes beyond four years are not available.

Risk of bias assessment identified methodological concerns in two of the three trials (Supplementary Figure S1). Inagaki et al. [10] was rated as high risk of bias, primarily due to selective outcome reporting of multiple categorical HbA1c thresholds not prespecified in the original trial registration, and substantial missing outcome data with differential attrition between treatment groups. These concerns are consequential given that Inagaki et al. [10] contributed approximately 49% of statistical weight to the glycemic outcomes analysis and was the sole source of weight loss data. Frias et al. [11] raised some concerns due to missing outcome data and imbalanced discontinuation patterns. Only Nicholls et al. [9] which dominated safety analyses, demonstrated consistently low risk of bias across all domains. The high risk of bias in trials contributing substantially to efficacy outcomes limits confidence in the magnitude of observed glycemic and weight loss benefits, though directionality aligns with broader indirect evidence syntheses.

Time-course data for adverse events and efficacy outcomes were not available in formats suitable for quantitative synthesis across trials; the kinetics of gastrointestinal tolerability during dose escalation which likely drive early discontinuation. Therefore, this could not be modeled, and the pooled estimates reflect cumulative incidence at trial end rather than temporal patterns of harm or benefit.

### 4.13. Future Research Directions

Future randomized controlled trials and evidence syntheses should prioritize: (1) standardized outcome definitions, particularly HbA1c < 7.0% as primary glycemic target and ≥5% weight reduction as primary weight threshold, to reduce analytical heterogeneity; (2) harmonized reporting of continuous outcomes with consistent timepoints and complete variance data to enable mean difference meta-analyses; (3) granular safety reporting including hypoglycemia by severity grade, gastrointestinal adverse events by subtype, and reasons for discontinuation; (4) follow-up durations of at least two years to assess durability, cumulative safety signals, and real-world persistence; and (5) patient-centered outcomes including quality of life, treatment satisfaction, and cost-effectiveness to inform shared decision-making [20,22].

Head-to-head trials should ensure that comparator doses reflect contemporary international standards (dulaglutide 1.5 mg as minimum; higher doses where regulatory approvals exist) to provide clinically relevant comparisons rather than regionally limited doses. Network meta-analyses incorporating indirect comparisons across the broader incretin landscape, including semaglutide 2.0–2.4 mg and emerging triple agonists, would provide a more comprehensive evidence base for positioning tirzepatide within the evolving therapeutic landscape for T2DM and obesity [21,22].

## 5. Conclusions

In this systematic review and meta-analysis of three randomized controlled trials (N = 13,590), overall adverse event incidence—the primary outcome—did not differ significantly between tirzepatide 15 mg and dulaglutide (RR 1.04, 95% CI: 0.98–1.10; moderate-certainty evidence). Treatment discontinuation due to adverse events was consistently higher with tirzepatide across all three included populations (RR 1.32, 95% CI: 1.20–1.45; I^2^ = 0%; high-certainty evidence), corresponding to a number needed to harm of approximately 32 over four years and representing the most robust and clinically actionable finding in this analysis.

Glycemic efficacy findings were highly heterogeneous and population dependent. Tirzepatide demonstrated numerically greater HbA1c reductions across all three trials, but magnitude and clinical interpretation differ substantially. At the primary threshold (HbA1c < 7.0%), tirzepatide was consistently superior with no heterogeneity (RR 1.48 [1.33–1.64]; I^2^ = 0%), providing the most reliable efficacy estimate in this analysis. Across stricter thresholds, heterogeneity was extreme (I^2^ = 92%), and the overall pooled estimate is not generalizable. The advantage was most pronounced in treatment-naive Japanese patients receiving the Japan-approved comparator dose (0.75 mg dulaglutide), more modest but still present in patients with established cardiovascular disease receiving standard-dose dulaglutide (1.5 mg; difference −0.78 pp, 95% CI: −0.84 to −0.72 at 36 months). Metabolic superiority in Nicholls et al. [9] trial did not translate into cardiovascular superiority, reinforcing that treatment selection should be guided by patient-specific priorities rather than assuming universal benefit from the more potent agent. Serious adverse event rates were not significantly different between treatments. Weight loss analyses favored tirzepatide, though these findings are limited by single-trial dominance and methodological constraints of the weight loss analysis.

These findings are broadly consistent with contemporary evidence syntheses indicating superior glycemic and weight efficacy with tirzepatide relative to GLP-1 receptor agonist-based strategies [15,16,17,18,19,20], while identifying treatment tolerability and persistence as the principal limiting factor for real-world benefit. Access and affordability further constrain the generalizability of comparative efficacy data in most health systems where tirzepatide is not reimbursed.

Tirzepatide may be favored when greater glycemic or weight efficacy is prioritized in patients with earlier-stage disease, treatment-naive status, or lower baseline BMI, provided gastrointestinal adverse effects are managed proactively and discontinuation risk is discussed before initiation. Dulaglutide remains a well-established option with proven cardiovascular benefit and may be preferred when treatment persistence is threatened by tolerability concerns, when cardiovascular risk reduction is the predominant therapeutic goal, or when access and cost are limiting factors. The optimal therapy is ultimately the one that individual patients can tolerate and persist with over time, underscoring the importance of shared decision-making that balances efficacy expectations against tolerability trade-offs, patient preferences, and real-world access.

## Figures and Tables

**Table 1:** Characteristics of included studies.

Study	Trial Name	Design	Duration	Location	Participants (N)	Intervention	Comparator	Primary Outcome	Risk of Bias
Inagaki et al. 2022 [10]	SURPASS J-mono	Double-blind RCT	52 weeks	Japan (multiple sites)	Tirz: 159; Dulg: 159	Tirzepatide 15 mg SC weekly	Dulaglutide 0.75 mg SC weekly	HbA1c change from baseline	High risk
Frias et al. 2018 [11]	Phase 2b (LY3298176)	Double-blind RCT	26 weeks	International (multiple countries)	Tirz: 53; Dulg: 54	Tirzepatide 15 mg SC weekly	Dulaglutide 1.5 mg SC weekly	HbA1c change from baseline	Some concerns
Nicholls et al. 2025 [9]	SURPASS-CVOT	Double-blind, event-driven RCT	Median 4.0 years (208 weeks)	30 countries, 640 sites	Tirz: 6586; Dulg: 6579	Tirzepatide 15 mg SC weekly	Dulaglutide 1.5 mg SC weekly	3-point MACE (CV death, MI, stroke)	Low risk

Abbreviations: CV, cardiovascular; Dulg, dulaglutide; MACE, major adverse cardiovascular events; MI, myocardial infarction; RCT, randomized controlled trial; SC, subcutaneous; Tirz, tirzepatide. Risk of bias was assessed using the Cochrane RoB 2 tool. High risk in Inagaki et al. [10] reflects concerns regarding post hoc reporting of efficacy thresholds and missing data.

**Table 2:** Baseline characteristics of study participants.

Characteristic	Inagaki et al. [10]		Frias et al. [11]		Nicholls et al. [9]	
	Tirz 15mg (N = 159)	Dulg 0.75mg (N = 159)	Tirz 15mg (N = 53)	Dulg 1.5mg (N = 54)	Tirz 15mg (N = 6586)	Dulg 1.5mg (N = 6579)
Demographics						
Age (years), mean ± SD	56.3 ± 10.2	56.3 ± 10.5	56.6 ± 9.1	56.6 ± 9.8	64.0 ± 8.8	64.1 ± 8.7
Female, n (%)	29 (18.2)	30 (18.9)	31 (58.5)	32 (59.3)	1891 (28.7)	1926 (29.3)
Race/Ethnicity, n (%)						
Asian	159 (100)	159 (100)	NR	NR	NR	NR
White	0 (0)	0 (0)	NR	NR	NR	NR
Hispanic/Latino	0 (0)	0 (0)	22 (41.5)	25 (46.3)	~1976 (30.0)	~1974 (30.0)
Anthropometrics						
Weight (kg), mean ± SD	72.8 ± 14.2	73.5 ± 15.1	93.2 ± 19.5	94.1 ± 20.2	92.6 ± 18.9	92.5 ± 18.8
BMI (kg/m^2^), mean ± SD	27.5 ± 3.8	28.0 ± 4.1	33.1 ± 5.8	34.4 ± 6.2	32.6 ± 5.5	32.6 ± 5.5
Glycemic Parameters						
HbA1c (%), mean ± SD	8.16 ± 0.85	8.25 ± 0.89	8.13 ± 1.02	8.09 ± 0.98	8.40 ± 0.92	8.38 ± 0.93
DM Duration (yrs)	5.0 ± 4.2	5.1 ± 4.5	8.6 ± 6.1	8.6 ± 6.3	14.8 ± 8.8	14.7 ± 8.7
Cardiovascular						
Systolic BP (mmHg)	129.8 ± 13.2	130.5 ± 14.1	130.2 ± 14.8	131.1 ± 15.2	135.1 ± 15.5	135.5 ± 15.8
History of ASCVD (%)	0 (0)	0 (0)	0 (0)	0 (0)	6586 (100)	6579 (100)
Renal Function						
eGFR (mL/min/1.73 m^2^)	91.9 ± 18.5	89.2 ± 19.8	90.5 ± 20.1	88.7 ± 21.3	78.5 ± 24.2	79.2 ± 23.5

Abbreviations: ASCVD, atherosclerotic cardiovascular disease; BMI, body mass index; BP, blood pressure; eGFR, estimated glomerular filtration rate; NR, not reported; SD, standard deviation. Nicholls et al. [9] enrolled a 100% secondary prevention population; Inagaki et al. (2022) [10] enrolled exclusively Japanese participants.

**Table 3:** Summary of reported continuous HbA1c and weight change outcomes.

Study	Timepoint	Tirzepatide Dose	Dulaglutide Dose	n (T/D)	Mean Change (T)	Mean Change (D)	Effect Estimate	CI/SE
HbA1c change								
Frias [11]	26 weeks	15 mg	1.5 mg	53/54	−1.94	−1.21	−0.73	80% CI: −0.95 to −0.52
Inagaki [10]	52 weeks	15 mg	0.75 mg	160/159	−2.8	−1.3	−1.5	SE: 0.1 (both arms)
Weight change								
Frias [11]	26 weeks	15 mg	1.5 mg	53/54	NR	−2.7 kg	−6.2 kg	NR
Inagaki [10]	52 weeks	15 mg	0.75 mg	160/159	−10.7 kg (−13.9%)	−0.5 kg (−0.7%)	−10.2 kg	SE: 0.4 (both arms)

Abbreviations: CI, Confidence Interval; D, Dulaglutide; SE, Standard Error; T, Tirzepatide.

**Table 4:** Summary of meta-analysis results and certainty of evidence (GRADE).

Outcome	Studies	N	RR (95% CI)	*p*-Value	I^2^ (%)	Certainty (GRADE)
Primary Safety						
Overall Adverse Events	3	13,720	1.04 (0.98–1.10)	0.24	36%	⊕⊕⊕◯ (Moderate)
Secondary Safety						
Discontinuation due to AEs	3	13,720	1.32 (1.20–1.45)	<0.001	0%	⊕⊕⊕⊕ (High)
Serious Adverse Events	3	13,710	0.82 (0.47–1.43)	0.49	42%	⊕⊕◯◯ (Low)
Efficacy						
HbA1c Target Achievement *	2	1274	1.95 (1.18–2.11)	0.001	92%	⊕◯◯◯ (Very Low)
Weight Loss Thresholds *	2	1275	8.80 (4.04–19.17)	<0.001	80%	⊕◯◯◯ (Very Low)

Notes: Abbreviations: AEs, adverse events; CI, confidence interval; GRADE, Grading of Recommendations, Assessment, Development and Evaluations; I^2^, inconsistency statistic; RR, risk ratio. * Efficacy pooled estimates are meta-analytic summaries for reference; multiple thresholds per trial are presented separately in forest plots. See Section 3.8 for GRADE downgrading rationale.

## Data Availability

The data is included in the manuscript and the attached Supplementary File.

## Supplementary Materials

The following supporting information can be downloaded at: https://www.mdpi.com/article/10.3390/healthcare14070850/s1, Table S1. PRISMA 2020 Checklist. Table S2. Characteristics of Excluded Studies. Table S3. Summary of Risk of Bias Assessment Using Cochrane RoB 2 Tool. Table S4. Data Accounting Table for Pooled Safety Outcomes. Table S5. Availability of Variance Data for Continuous Outcomes. Figure S1. Risk of Bias Summary Plot. Figure S2. Funnel Plot: Overall Adverse Events. Figure S3. Funnel Plot: Discontinuation due to AEs. Figure S4. Funnel plot for serious adverse events. Figure S5. Funnel Plot: HbA1c Target Achievement. Figure S6. Funnel plot for weight loss outcomes. References [23,24,25,26] are cited in the Supplementary Materials.
