# Supplementary material for: Comparative Efficacy and Safety of Tirzepatide Versus Dulaglutide in Patients with Type 2 Diabetes Mellitus: A Systematic Review and Meta-Analysis
Source: Healthcare (Basel). 2026 Mar 27;14(7):850. doi: 10.3390/healthcare14070850 (PMC13073533; doi:10.3390/healthcare14070850)

Comparative Efficacy and Safety of Tirzepatide versus Dulaglutide in Patients with Type 2 Diabetes Mellitus: A Systematic Review and Meta-Analysis

Supplementary Table S1: PRISMA 2020 Checklist

Registration: PROSPERO CRD420251276594

#	Item	Page	Location in Manuscript
**TITLE**			
1	Identify report as systematic review	1	Title states "Systematic Review and Meta-Analysis"
**ABSTRACT**			
2	Provide structured summary	1-2	Abstract includes Background, Methods (with N=13,590 participants), Results, Conclusion
**INTRODUCTIO N**			
3	Describe rationale	2-3	Introduction paragraphs 1-6
4	Provide explicit objectives	3	Final paragraph: "The primary objective is to assess overall adverse event incidence..."
**METHODS**			
5	Eligibility criteria	4	Section 2.3: Inclusion/exclusion criteria with intervention/comparator definitions
6	Informatio n sources	3-4	Section 2.2: MEDLINE, Embase, Scopus, CENTRAL; inception–December 2025
7	Search strategy	4	*Section 2.2:* Search Strategies by Database
8	Selection process	4	Section 2.5: Two reviewers independently using Rayyan, consensus
9	Data collection	4	Section 2.6: Two reviewers, standardized pre-piloted form
10a	Data items - outcomes	4-5	Section 2.4: Primary, secondary safety, and efficacy outcomes
10b	Data items - other variables	4	Section 2.6: Study and participant characteristics listed
11	Risk of bias assessment	5	Section 2.7: Two reviewers, RoB 2 tool
12	Effect measures	5	Section 2.9: Risk ratios (RRs) with 95% CIs
13a	Synthesis methods - eligibility	5	Sections 2.4.4 and 2.9

13b	Synthesis methods - data preparation	4-5	Sections 2.6 and 2.4.4: n/N format extraction
13c	Synthesis methods - display	5, 9-14	Section 2.9 describes forest plots; Figures 2-6, Tables 1-3
13d	Synthesis methods - models	5	Section 2.9: Random-effects Mantel-Haenszel, I^2 statistic, RevMan 5.4
13e	Heterogeneity exploration	5	Section 2.10: Subgroup by dulaglutide dose (0.75 vs 1.5 mg)
13f	Sensitivity analyses	5	Section 2.10: Dose-stratified subgroup analyses
14	Reporting bias assessment	5-6	Section 2.11: Funnel plots for outcomes with ≥3 studies
15	Certainty assessment	5	Section 2.8: GRADE approach
**RESULTS**			
16a	Study selection	6-7	Section 3.1 with numbers; Figure 1 PRISMA flowchart
16b	Excluded studies	6	Section 3.1: Four excluded (reasons stated); Supplementary Table S3
17	Study characteristics	7-9	Section 3.2 (Table 1); Section 3.3 (Table 2)
18	Risk of bias	7	Section 3.2: "Supplementary Figure S1, Table S3"
19	Individual study results	9-13	Figures 2-6 show each study's RR and 95% CI; Sections 3.4-3.6
20a	Synthesis characteristics	9-14	Sections 3.4-3.6; Table 3
20b	Synthesis results	9-14	All meta-analyses: RR, 95% CI, p-values, I^2; Figures 2-6; Table 3
20c	Heterogeneity causes	11-12	Section 3.6.1: Subgroups by dose/population; Sections 3.6.1.1-3
20d	Sensitivity analyses	11-13	Dose-stratified results for glycemic/weight outcomes
21	Reporting bias	13-14	Section 3.7: Funnel plot assessments (subsections 3.7.1-5)
22	Certainty of evidence	14	Table 3: GRADE ratings (⊕⊕⊕⊕ to ⊕◯◯◯); Section 3.8
**DISCUSSION**			

23a	General interpretation	14-21	Sections 4.1-4.11; Section 4.3.3 "Contextualization"
23b	Limitations of evidence	21-22	Section 4.12
23c	Limitations of review	21-22	Section 4.12
23d	Implications	18-23	Sections 4.8-4.11 (practice); Section 4.13 (research)
**OTHER**			
24a	Registration	1, 3	Abstract and Section 2.1: PROSPERO CRD420251276594
24b	Protocol access	3	Section 2.1: https://www.crd.york.ac.uk/prospero/display_record.php?ID=CRD420251276594
24c	Amendments	3	Section 2.1: "No amendments were made"
25	Support	24	Funding: No funding; APC by Alfaisal University
26	Competing interests	24	Conflicts of Interest: No conflicts
27	Data availability	24	Data Availability: Included in manuscript and supplementary files

Supplementary Table S2: Characteristics of Excluded Studies

Study Title	Citation	Reason for Exclusion
Evaluation of Insulin Dosage After the Addition of Tirzepatide Compared With Semaglutide or Dulaglutide in Patients With Type 2 Diabetes	McKone DB, Duprey KS, Hall HM, Leonhard A, Schadler A, Naseman KW. Diabetes Spectr. 2025;38(3):266-273. doi: 10.2337/ds24-0035	Dual population study (included both semaglutide and dulaglutide comparisons; no separate tirzepatide vs dulaglutide data extractable)
Comparative Gastrointestinal Safety of Dulaglutide, Semaglutide, and Tirzepatide in Patients with Type 2 Diabetes	Crisafulli S, Alkabbani W, Paik JM, Bykov K, Tavakkoli A, Glynn RJ, et al. Ann Intern Med. 2026;179(1):1-11. doi: 10.7326/ANNALS-25-01724. Epub 2025 Nov 4	Not reporting outcomes of interest (focused only on gastrointestinal safety endpoints; did not report overall adverse events, discontinuation, serious adverse events, or efficacy outcomes specified in protocol)
Emulation of the Study of Tirzepatide Compared with Dulaglutide on Major Cardiovascular Events in	John Ostrominski, Janinne Ortega-Montiel, Helen Tesfaye, Caroline Alix, Deborah J. Wexler, Julie M. Paik, Elisabetta Patorno; 223-OR:	LNCT06779929

Participants with Type 2 Diabetes (SURPASS-CVOT)	Emulation of the Study of Tirzepatide Compared with Dulaglutide on Major Cardiovascular Events in Participants with Type 2 Diabetes (SURPASS-CVOT). Diabetes 20 June 2025; 74 (Supplement_1): 223–OR. https://doi.org/10.2337/db25-223-OR	
Change in pharmacodynamic variables following once-weekly tirzepatide treatment versus dulaglutide in Japanese patients with type 2 diabetes (SURPASS J-mono substudy)	Yabe D, Kawamori D, Seino Y, Oura T, Takeuchi M. Diabetes Obes Metab. 2023;25(2):398-406. doi: 10.1111/dom.14882	Duplicate population (substudy of SURPASS J-mono trial already included as **Inagaki** et al. 2022; focused on pharmacodynamic variables rather than prespecified clinical outcomes)

Notes:

1. **Dual population study:** Study compared tirzepatide with multiple comparators (both semaglutide and dulaglutide) without providing separate extractable data for the tirzepatide vs dulaglutide comparison.
2. **Not reporting outcomes of interest:** Study focused exclusively on gastrointestinal safety endpoints and did not report the prespecified outcomes for this systematic review (overall adverse events, treatment discontinuation due to adverse events, serious adverse events, glycemic control targets, or weight loss thresholds).
3. **Incorrect study design:** Study was an observational emulation/simulation study rather than a randomized controlled trial, which was required per the eligibility criteria (Section 2.3).
4. **Conference abstract only:** Study was identified only as a conference abstract or substudy report without a full-text peer-reviewed manuscript available for comprehensive quality assessment and data extraction.

Supplementary Table S3. Summary of Risk of Bias Assessment Using Cochrane RoB 2 Tool

Study	D1: Randomization Process	D2: Deviations from Intended Interventions	D3: Missing Outcome Data	D4: Measurement of Outcome	D5: Selection of Reported Result	Overall Judgment
**Nicholls et al. 2025**	⊕ Low risk	⊕ Low risk	⊕ Low risk	⊕ Low risk	⊕ Low risk	**⊕ Low risk**
**Frias et al. 2018**	⊕ Low risk	⊕ Low risk	⊖ Some concerns	⊕ Low risk	⊕ Low risk	**⊖ Some concerns**

Inagaki et al. 2022	⊕ Low risk	⊕ Low risk	⊖ Some concerns	⊕ Low risk	⊖ High risk	**⊖ High risk**

Legend:
⊕ Low risk of bias | ⊖ Some concerns | ⊖ High risk of bias

Supplementary Table S4: Data Accounting Table for Pooled Safety Outcomes

Trial / Study	Overall Adverse Events (n/N)	Treatment Discontinuation (n/N)	Serious Adverse Events (n/N)	Population Type (Primary Source)
**Frias et al. 2018**	**Tirz:** 45 / 53<br>**Dulg:** 40 / 54	**Tirz:** 13 / 53<br>**Dulg:** 6 / 54	**Tirz:** 2 / 53<br>**Dulg:** 3 / 54	**Safety Set:** All participants who received at least one dose of study medication.
**Nobuya Inagaki et al. 2022**	**Tirz:** 134 / 160<br>**Dulg:** 123 / 159	**Tirz:** 16 / 160<br>**Dulg:** 9 / 159	**Tirz:** 7 / 160<br>**Dulg:** 14 / 149*	**Modified ITT:** All randomized participants who received ≥1 dose and had ≥1 post-baseline safety assessment.
**Nicholls et al. 2025**	**Tirz:** 5956 / 6647<br>**Dulg:** 5894 / 6647	**Tirz:** 878 / 6647<br>**Dulg:** 672 / 6647	**Tirz:** 2217 / 6647<br>**Dulg:** 2121 / 6647	**Intention-to-Treat (ITT):** All randomized participants regardless of treatment adherence.
**Pooled Totals**	**Tirz:** 6135 / 6860<br>**Dulg:** 6057 / 6860	**Tirz:** 907 / 6860<br>**Dulg:** 687 / 6860	**Tirz:** 2226 / 6860<br>**Dulg:** 2138 / 6850*	—

Abbreviations: Dulg, Dulaglutide; ITT, Intention-to-Treat; Tirz, Tirzepatide.

Note (*): Denominator variation in the Inagaki et al. SAE arm (149 vs. 159) reflects the specific safety-evaluable population for serious events reported in the primary trial results.

Supplementary Table S5: Availability of Variance Data for Continuous Outcomes

Study / Trial	Primary Timepoint	HbA1c: Format of Reported Data	Body Weight: Format of Reported Data	Reason for Exclusion from Pooled Meta-Analysis
**Phase 2b (LY3298176)** (Frias et al. 2018)	26 weeks	Least square mean (80% Credible Interval)	Mean Change (Units: kg) (No variance measure reported)	Differing follow-up duration (26 weeks); use of Credible Intervals (Bayesian) instead of SD/SE prevents standard frequentist pooling.
**SURPASS J-mono** (Inagaki et al. 2022)	52 weeks	Least square mean (Standard Error)	Least square mean (Standard Error)	Variance reported as SE for LS means at 52 weeks; incompatible with the years-long data from the CVOT.
**SURPASS-CVOT** (Nicholls et al. 2025)	36 months (median)	95% Confidence Interval (Percentage Points)	95% Confidence Interval (Units: kg)	Significant clinical heterogeneity in follow-up duration (36 months vs. 26/52 weeks) violates requirements for continuous pooling.

Supplementary Figures

Supplementary Figure S1. Risk of Bias Summary Plot

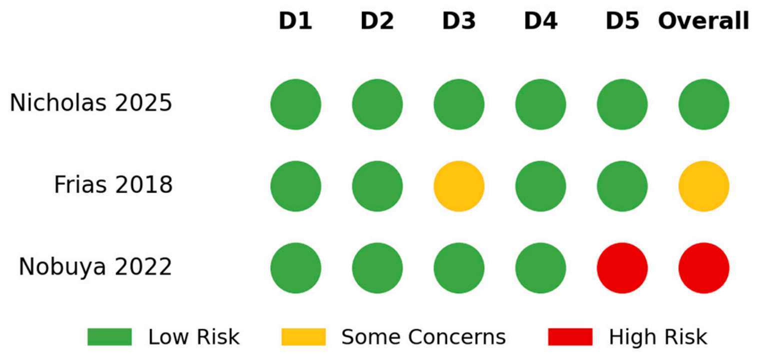

Note: Risk of bias assessment for included randomized controlled trials using the Cochrane Risk of Bias 2 (RoB 2) tool. Each trial was evaluated across five domains: randomization process (D1), deviations from intended interventions (D2), missing outcome data (D3), measurement of the outcome (D4), and selection of the reported result (D5). Overall risk of bias judgments were derived using the RoB 2 algorithm.

Supplementary Figure S2. Funnel Plot: Overall Adverse Events

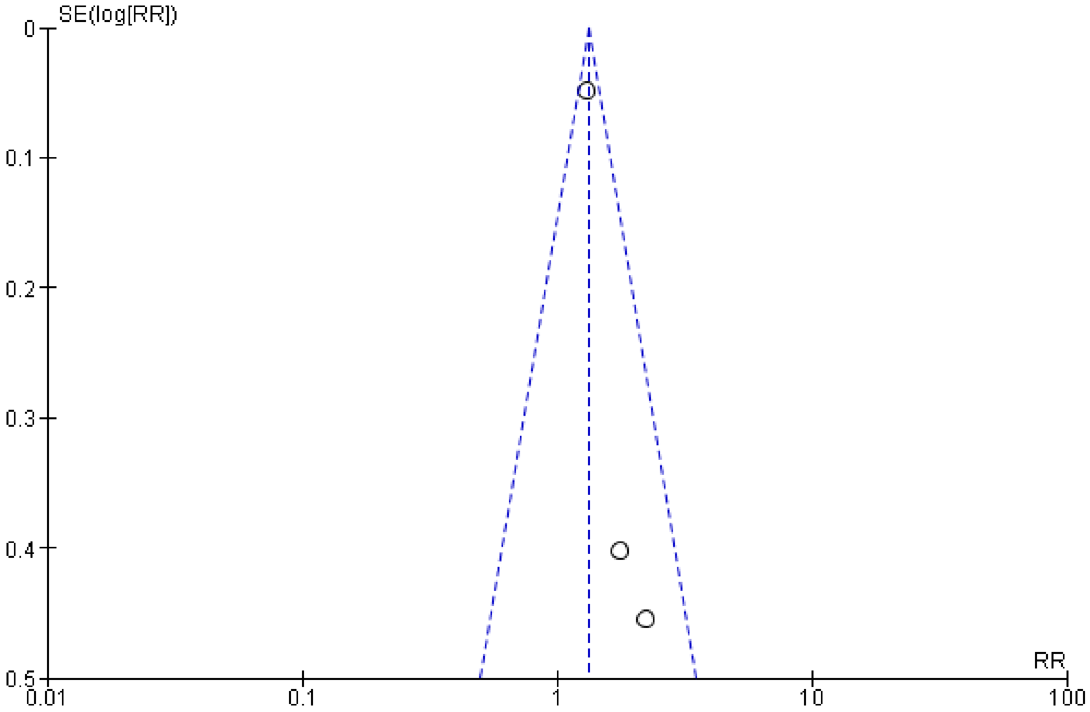

Supplementary Figure S3.Funnel Plot: Discontinuation due to AEs

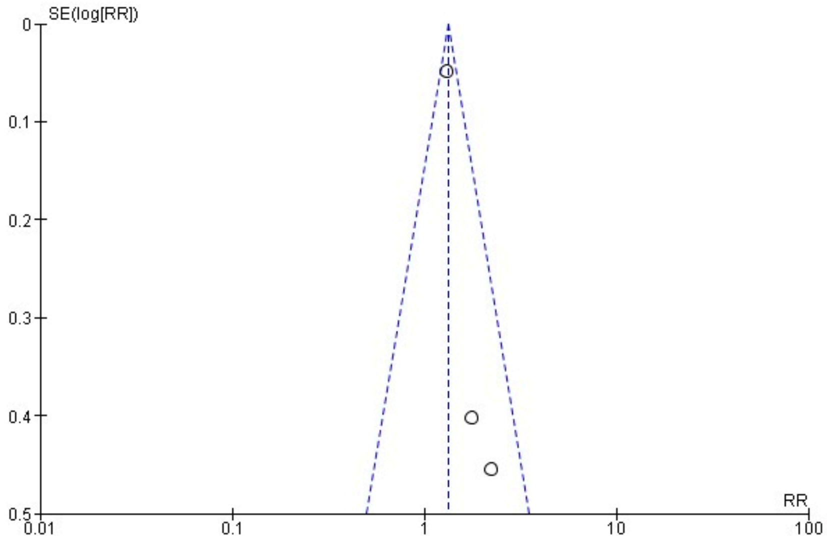

Supplementary Figure S4. Funnel plot for serious adverse events.

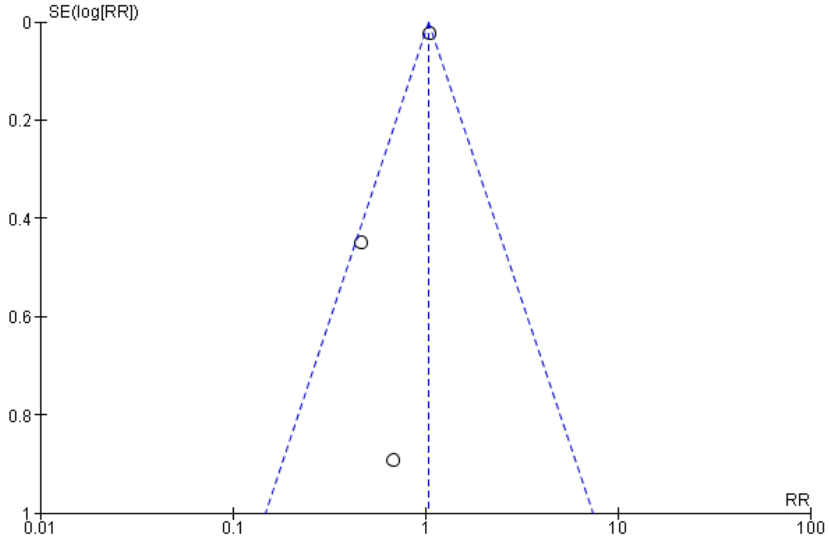

Supplementary Figure S5. Funnel Plot: HbA1c Target Achievement

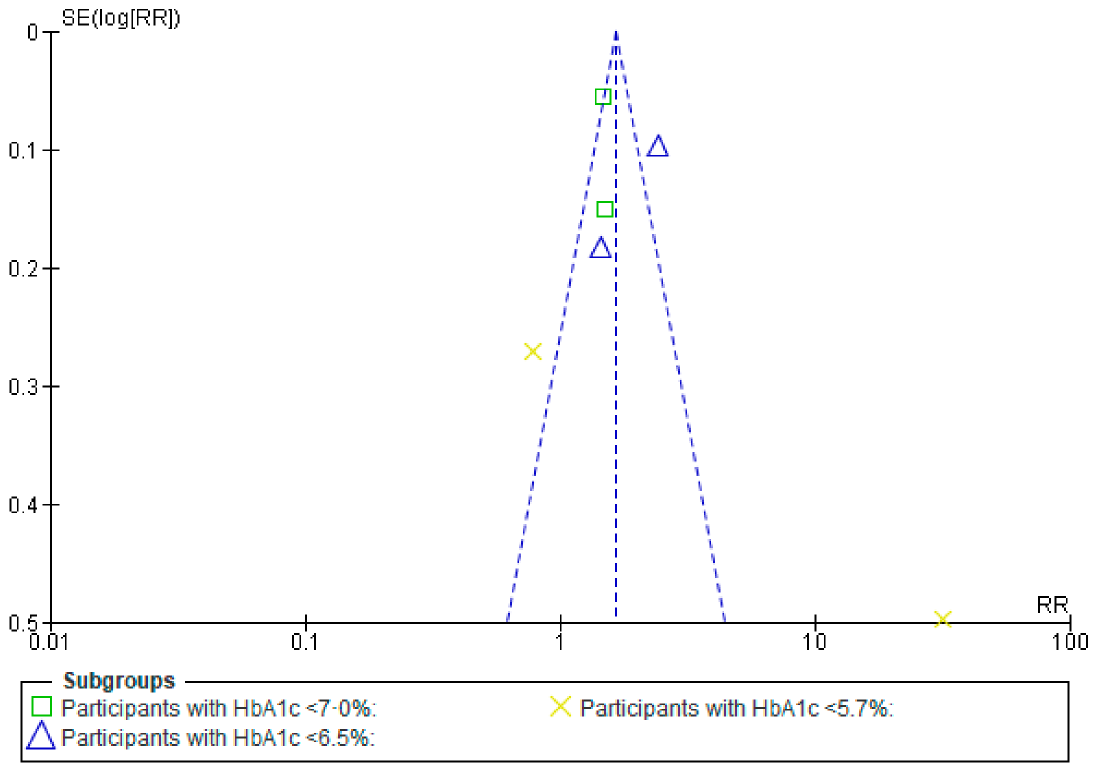

Supplementary Figure S6. Funnel plot for weight loss Outcomes

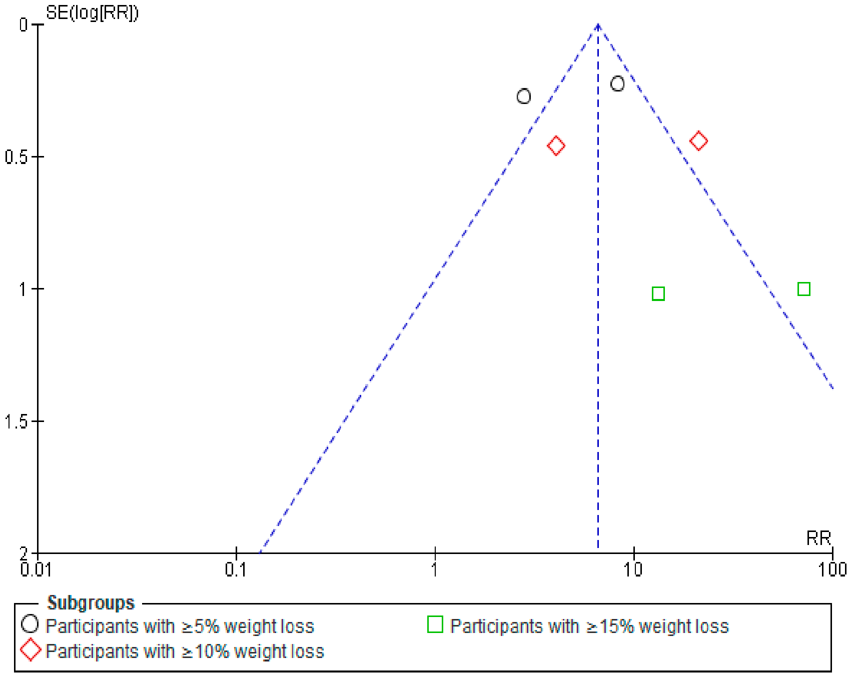

Supplement: Supplementary file 1 [file healthcare-14-00850-s001.zip › healthcare-4174566-supplementary.pdf]
